# Habitual physical activity is related to more creative activities and achievements

**DOI:** 10.1038/s41598-024-80714-6

**Published:** 2024-11-30

**Authors:** Christian Rominger, Andreas Fink, Corinna M. Perchtold-Stefan, Mathias Benedek, Andreas R. Schwerdtfeger

**Affiliations:** University of Graz, Graz, Austria

**Keywords:** Bodily movement, Real-life creativity, Creative achievements, Psychology, Human behaviour

## Abstract

Beyond health, physical activity (PA) is associated with better creative ideation performance. People who are habitually more physically active produce more original ideas when asked to think creatively about an alternative use of an everyday object such as a brick. This aligns with findings indicating a positive relationship of PA with more basic cognition, such as memory, attention, as well as executive functioning. However, to the best of our knowledge, to date, no empirical research examined the link between PA (or inactivity) and real-life creative behavior. Therefore, this study investigated how objective and subjective assessments of PA relate to creative activities and achievements. In a sample of 156 participants wearing a mobile sensor for up to five consecutive days, less sedentary behavior and more moderate-to-vigorous PA were associated with more frequent creative activities. A similar pattern of associations occurred for self-rated habitual PA, which explained additional and unique variance of creative activities as well as creative achievements. For objective PA (assessed via sensor), we did not find associations with creative achievements, openness, and creative behavior. The results of this study suggest that the positive effects of habitual PA may generalize from better health, cognition, and creative ideation performance outcomes to more real-life creative behavior such as cooking original dishes and making music.

**Supplementary Information:**

The online version contains supplementary material available at 10.1038/s41598-024-80714-6.

## Introduction

Physical activity (PA) is associated with increased life expectation^1,2^. In line with this, a sedentary lifestyle seems to increase mortality^3^. Beyond these effects of PA on quantitative aspects of life, PA also impacts the quality of people’s life in terms of better sleep^4^, reduced depressive symptoms^5,6^, and more well-being (^7^; but see^8^). Furthermore, PA is associated with better cognitive functioning such as increased working memory, attentional skills, and executive functions in (older) adults as well as in children (^9–13^; but see e.g.,^14^). These positive effects of PA on basic cognition seem to generalize to more complex cognitive functions such as creative ideation performance^15^ and even more broadly, to real-life academic achievements in children and adolescents (^16^; for moderation effect see^17,18^).

However, available studies on (creative) cognition are often challenged by methodological shortcomings such as that both intervention studies and self-report studies could be biased by expectation effects regarding the studied effects of PA^19,20^. Passive monitoring of PA via wearables might constitute one potential solution for this problem (see e.g.,^18^). The application of this technology ensures double blinding in principle and therefore provide potentially less expectation-driven study results. In a sample of 79 participants, Rominger et al.^21^ used wearables throughout a measurement period of 5 consecutive days of a typical week to assess people’s habitual PA level. The findings indicated a positive association between creative ideation performance with the percentage of moderate activity and a negative association with sedentary behavior. Furthermore, the total amount of physical activity per minute (i.e., counts per minute) was significantly associated with people’s creative performance (see also,^22^). In a sample of more than 140 school-aged children, Romance et al.,^20^ found an association between moderate-to-vigorous PA and creativity assessed via the Torrance Test of Creative Thinking (TTCT;^23^). These studies suggest a positive relationship between objectively assessed (via accelerometry) habitual PA and creative potential. Accordingly, people who are physically more active produce more original ideas (for a meta-analysis of cross-sectional studies, see^15^). Importantly, these relationships do not indicate causality—therefore, it is also possible that creativity leads to more PA.

Although the associations between PA and cognition, academic achievements, as well as creative cognition abilities seem robust^13,16^, we do not know yet if habitual PA is also associated with real-life creative behavior such as creative activities and creative achievements. Hence, do people, who are habitually more physically active, also engage more often in creative activities such as writing blog entries, making up melodies or new recipes? Furthermore, do they produce more creative achievements such as artworks, publications or computer programs that get broader attention? So far, we know that creative potential especially in terms of openness to experience, creative self-beliefs, cognitive functioning (i.e., intelligence), and creative ideation ability predicts creative behavior^24,25^. Here, we asked whether PA is also related to real-life creativity and potentially explains additional variance. Therefore, the present study targeted to explore the PA—creative activity relationship by means of passive sensor technology in combination with a questionnaire assessing habitual PA in a sample of 156 young participants.

First, we hypothesized a positive association between objective PA (i.e., percentage of moderate-to-vigorous PA, sedentariness, number of steps) and creative activities as well as achievements. Second, we further expected a similar pattern for subjectively assessed habitual PA. To investigate the unique contribution of objective and subjective PA measures on real-life creativity, we calculated two regression analyses to predict creative activities and creative achievements, controlling for potential confounders such as age, gender, and BMI as well as well-grounded predictors of creative activities and achievements such as openness to experience, creative self-beliefs, and creative ideation ability.

## Methods

### Participants

In total, 156 participants (48 men) with a mean age of 23.74 years (*SD* = 4.61, min = 17, max = 54) and a mean BMI of 22.42 kg/m^2^ (*SD* = 3.58) took part in this study. The samples size was sufficient to detect a small to medium effect of *r* ≥ 0.22 with an Alpha of 0.05 and a power of 0.80, as expected according to a meta-analysis indicating on habitual PA and creative ideation performance^15^. According to self-report, all participants were free of cardiovascular, neurological, or mental disorders as well as psychotropic or cardiovascular medication. We recruited the participants via email and social media. The ethics review board of the University of Graz approved this study as a part of a larger project (GZ. 39/100/63 ex 2020/21). The methods were carried out in accordance with this, and written informed consent was obtained.

### Procedure

After giving informed consent, participants completed the online questionnaires (Limesurvey; Limesurvey GmbH, Hamburg, Germany). Then, they wore the acceleration sensor for (up to) five consecutive days from Wednesday to Sunday on their chest to record major bodily movements (daily from 09:00 to 21:00). Finally, participants returned the equipment and received financial compensation (30 Euros) or course credits.

### Creativity assessment

#### Real-life creative activities and creative achievements

We assessed creative activities (CAct) and creative achievements (CAch) with the Inventory of Creative Activities and Achievements (ICAA; 24). The IACC measures the frequency of creative activities and the significance of creative achievements across eight creative domains (i.e., literature, music, arts and crafts, cooking, sports, visual arts, performing arts, science, and engineering). For CAct, people reported on a five-point Likert scale how frequently a certain activity has been performed in the past 10 years from “never” to “more than 10 times” with six activities per domain. The internal consistency of single domain scores ranged between α = 0.67 and α = 0.82. The creative activities total score showed acceptable internal consistency (α = 0.76). The CAch subscale asks people to indicate the level of achievements in the same eight creative domains from “I have never been engaged in this domain” to “I have already sold some of my work in this domain”. In line with Diedrich et al.^24^, and because the levels of creative achievements are not independent, we did not calculate internal consistencies for each domain score. The total creative achievements score showed acceptable internal consistency despite the diversity of underlying domains (α = 0.71).

#### Self-reported ideational behavior (RIBS)

We used the short German version of Runco’s Ideational Behavior Scale (RIBS;^26^; see e.g.,^24^) to assess creative ideation behavior. It includes 17 statements such as “I come up with an idea or solution other people have never thought of”. Participants responded to the items on a scale ranging from 1 (never) to 5 (very often; *M* = 3.71, *SD* = 0.88; α = 0.92).

#### Creative ability in the figural domain (TCT-DP)

Participants completed the abstract picture fragments of the Test for Creative Thinking–Drawing Production online (TCT-DP;^27^). Participants sent the generated drawings to the experimenter or stored them online via limesurvey (Limesurvey; Limesurvey GmbH, Hamburg, Germany). Two independent and trained raters (one man and one woman) scored the TCT-DP in accordance with the test manual (e.g., unconventionality, inclusion of new elements, graphic combinations, etc.). Due to the online-application, we did not evaluate the time component of the TCT-DP. We used the mean score of both raters as an index of creative ability (*M* = 1.85, *SD* = 0.76). The interrater reliability was high (*r* = 0.94). Due to technical problems, five completed picture fragments were lost. We imputed these five cases via the R package missForest (vers. 1.5;^28^) by taking available information into account (i.e., gender, age, BMI, openness, self-reported ideational behavior, indices of objective and subjective PA).

#### Openness

We assessed openness via the NEO-FFI (^29^; German translation^30^). Openness is consistently associated with creative ideation performance, as well as real-life creative activities and creative achievements^24,31,32^. The internal consistency of the openness scale in the present study was good with α = 0.76 (*M* = 2.89, *SD* = 0.54).

### PA assessment

#### Self-rated subjective measurement of PA

The Freiburger Questionnaire on Physical Activity (FQPA;^33^) is a widely used self-assessment of PA (e.g.,^34–37^). The questionnaire covers every day physical activities (e.g., walking to work), leisure time activities (e.g., dancing), and sports-related activities (e.g., swimming). Participants reported the duration for each activity per week, which can be converted into metabolic equivalents (MET;^38^). Similar to previous work, we used the sum score of METs as a measure of habitual PA^36^. The mean of the total METs was 46.41 (*SD* = 34.69) per week. Frey et al.^33^ reported a two weeks re-test reliability of *r* = 0.95 for the total amount of PA.

#### Objective measurement of PA via ambulatory sensor

Participants wore the accelerometric sensor for five consecutive days from Wednesday to Sunday on their chest (from 09:00 to 21:00;^39^, for a similar approach see^21^). Prior to the assessment, participants received a brief introduction in the use of the sensor. The EcgMove4 (movisens GmbH, Karlsruhe, Germany) continuously recorded acceleration information with the build-in 3-dimensional accelero-sensor (64 Hz sampling rate) and a barometric sensor (8 Hz sampling rate). The easy-to-handle and small chest-worn device (62.3 mm × 38.6 × 11.5 mm) has a weight of 26 g. The sensor provides data on body positions, wear time, intensity of bodily movements, and number of steps.

#### Classification of intensity levels of PA by means of metabolic equivalent

We calculated the metabolic equivalent (MET) per minute as a measure of PA by means of the DataAnalyzer software (movisens GmbH, Karlsruhe, Germany; see also^40^). The movisens DataAnalyzer software calculates the MET in two steps. First, it estimates the activity class based on acceleration and barometric signals. Second, based on the detected activity class (i.e., sitting, walking, etc.) the algorithm choses the corresponding model for MET estimations, which takes the accelerometric score, the barometric data, and the personal parameters (i.e., age, gender, weight, height) into account.

By means of the calculated METs, we classified each minute of the 5 days recording into sedentary behavior (METs ≤ 1.5), physical activities of light-intensity (METs > 1.5 and ≤ 2.9) such as slow walking, moderate-to-vigorous intensity (METs > 2.9) such as fast walking, playing table tennis, running, hiking, or bicycling (e.g.,^38,41,42^; for similar classification see^40^). The DataAnalyzer automatically classifies the wear time (worn vs. non worn). We only included 1-min segments the sensor was worn to ensure validity of the activity classes. Table 1 reports the descriptive statistics for PA measures. The percentage of moderate-to-vigorous PA was the sum of 1-min segments in moderate-to-vigorous PA divided through the sum of valid minutes and multiplied by 100. We calculated sedentariness in analogy to this. Step per minute is the average number of steps of the total valid data.

**Table 1:** Descriptive statistics of PA variables.

Variable	*M*	*SD*	Min	Max	ICC(2,k)
Subjective PA					
Total METs	46.41	34.69	0	226.67	
Everyday activities METs	14.30	12.04	0.00	70.05	
Leisure time activities METs	11.93	13.01	0.00	72.00	
Sports activities METs	20.18	29.00	0.00	208.00	
Objective PA					
Steps per minute	8.77	3.91	0.76	23.11	0.67
Moderate-to-vigorous PA (%)	8.27	3.95	0.21	21.93	0.66
Sedentariness (%)	76.80	7.48	56.53	95.85	0.76

ICC(2,k) is the intraclass correlation coefficient to assess consistency of objective PA indices across the five days of measurement).

### Statistical analysis

We calculated Pearson correlations to analyze the zero-order associations between real-life measures of creativity with self-rated (i.e., total METs) and objective measures of PA (i.e., steps per minute, moderate-to-vigorous PA, sedentariness). To control for potential confounding variables and examine unique explained variance, we followed up these correlation analyses with regression analyses predicting real-life creativity via subjective PA (i.e., total METs) and objective PA (i.e., moderate-to-vigorous PA), controlling for gender, age, BMI, openness to experience, creative self-beliefs, and creative ideation ability. Finally, we explored relationships also at the level of creative domains by computing single order correlations for each domain subscale of the ICAA. All analyses were calculated in R (Version 4.4.1;^43^). We kept the level of significance fixed at *p* < 0.05 (two-tailed).

## Results

**Fig. 1:**
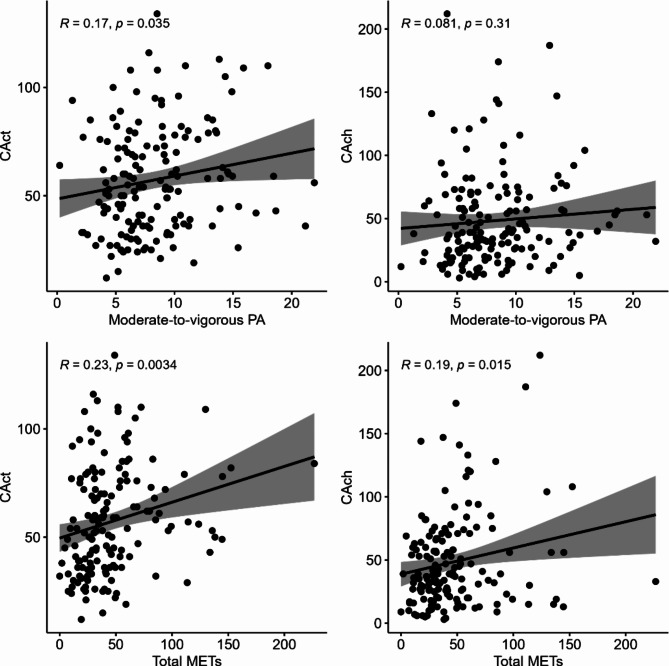
Pearson correlations between objective PA (i.e., moderate-to-vigorous PA) and subjective PA (i.e., total METs) with CAct and CAch.

As expected, objective PA measures were substantially correlated with higher moderate-to-vigorous PA relating to more steps and lower sedentariness (Table 2); yet, objective indicators of PA showed only modest associations with subjective PA. Similarly, measures of creative behavior showed expected associations with openness, self-reported ideational behavior (RIBS), and creative ability in the figural domain, demonstrating evidence of convergent validity. In line with our hypothesis, subjective PA was associated with higher creative activities and achievements (see Fig. 1). Importantly, higher engagement in creative activities was also significantly correlated to more steps per minute, lower sedentariness, and higher PA in moderate-to-vigorous intensity. Similar trends were observed for the other creativity measure, which however failed to reach statistical significance.

**Table 2:** Pearson correlations among variables.

	Sedentariness	Moderate-to-vigorous PA	Subjective PA (total METs)	CAct	CAch	Openness	RIBS	TCT-DP
*Steps*	-0.780 **(< 0.001)**	0.905 **(< 0.001)**	0.148 **(0.065)**	0.194 **(0.015)**	0.079 **(0.327)**	0.125 **(0.120)**	0.105 **(0.193)**	0.019 **(0.812)**
*Sedentariness*		-0.826 **(< 0.001)**	-0.211 **(0.008)**	-0.171 **(0.033)**	-0.093 **(0.250)**	-0.040 **(0.616)**	-0.074 **(0.360)**	0.038 **(0.635)**
*Moderate-to-vigorous PA*	-0.826 **(< 0.001)**		0.164 **(0.040)**	0.169 **(0.034)**	0.081 **(0.314)**	0.121 **(0.133)**	0.124 **(0.122)**	0.000 **(1.00)**
*Subjective PA (total METs)*	-0.211 **(0.008)**	0.164 **(0.040)**		0.233 **(0.003)**	0.194 **(0.015)**	0.109 **(0.177)**	0.061 **(0.447)**	-0.064 **(0.428)**
*CAct*	-0.171 **(0.033)**	0.169 **(0.034)**	0.233 **(0.003)**		0.555 **(< 0.001)**	0.379 **(< 0.001)**	0.399 **(< 0.001)**	0.163 **(0.042)**
*CAch*	-0.093 **(0.250)**	0.081 **(0.314)**	0.194 **(0.015)**	0.555 **(< 0.001)**		0.301 **(< 0.001)**	0.372 **(< 0.001)**	0.262 **(0.001)**
*Openness*	-0.040 **(0.616)**	0.121 **(0.133)**	0.109 **(0.177)**	0.379 **(< 0.001)**	0.301 **(< 0.001)**		0.533 **(< 0.001)**	0.301 **(< 0.001)**
*RIBS*	-0.074 **(0.360)**	0.124 **(0.122)**	0.061 **(0.447)**	0.399 **(< 0.001)**	0.372 **(< 0.001)**	0.533 **(< 0.001)**		0.217 **(0.007)**
*TCT-DP*	0.038 **(0.635)**	0.000 **(1.00)**	-0.064 **(0.428)**	0.163 **(0.042)**	0.262 **(0.001)**	0.301 **(< 0.001)**	0.217 **(0.007)**	

*p* values are in parentheses and are bold if significant. PA METs = Sum of METs per week assessed via FQPA, Creative achievements = CAch, Creative activities = CAct, Runco’s Ideational Behavior Scale = RIBS. TCT-DP = Test for Creative Thinking–Drawing Production.

The regression analysis with creative activities as the outcome variable was significant (*F*(8,147) = 9.631, *p* < 0.001, *R*^*2*^ = 0.344) indicating that moderate-to-vigorous activity predicted creative activities (B = 0.97, *t* = 2.193, *p* = 0.030) as well as subjective PA (B = 0.13, *t* = 2.650, *p* = 0.009) over and above all other variables. The effect of gender was also significant, indicating more creative activities for women than men (B = -16.47, *t* = -4.320, *p* < 0.001). Age (B = -0.35, *t* = -0.957, *p* = 0.340) and BMI (B = -0.54, *t* = -1.151, *p* = 0.251) were not significant. Furthermore, with respect to the creativity relevant predictors, only RIBS (B = 10.04, *t* = 4.349, *p* < 0.001) added independent and unique variance to the prediction model, but not the TCT-DP (B = 3.07, *t* = 1.322, *p* = 0.188) and openness (B = 5.330, *t* = 1.399, *p* = 0.164; see Table 3).

**Table 3:** Results of the two regression analyses predicting CAct and CAch.

Predictors	CAct			CAch		
	Estimates	CI (95%)	p-value	Estimates	CI (95%)	p-value
Intercept	10.29	-24.58 to 45.16	0.561	-65.10	-120.50 to -9.70	**0.022**
Gender	-16.47	-24.01 to -8.94	** < 0.001**	-20.04	-32.01 to -8.07	**0.001**
Age	-0.35	-1.06 to 0.37	0.340	0.04	-1.10 to 1.18	0.942
BMI	-0.54	-1.47 to 0.39	0.252	1.06	-0.42 to 2.54	0.159
Openness	5.33	-2.20 to 12.86	0.164	3.52	-8.44 to 15.49	0.561
RIBS	10.04	5.48 to 14.60	** < 0.001**	13.65	6.40 to 20.90	** < 0.001**
TCT-DP	3.07	-1.52 to 7.67	0.188	10.92	3.62 to 18.22	**0.004**
Objective PA (Moderate-to-vigorous)	0.97	0.10 to 1.84	**0.030**	0.69	-0.70 to 2.07	0.328
Subjective PA (total METs)	0.13	0.03 to 0.23	**0.009**	0.18	0.02 to 0.33	**0.025**
Observations	156			156		
R^2^ / R^2^ adjusted	0.344/0.308			0.271/0.232		

Gender was coded 0 = women and 1 = men. BMI = body mass index.

Significant p-values are bold.

The regression analyses with creative achievements as outcome variable was also significant (*F*(8,147) = 6.838, *p* < 0.001, *R*^*2*^ = 0.271), indicating that the total amount of self-rated METs predicted creative achievements (B = 0.18, *t* = 2.262, *p* = 0.025), but moderate-to-vigorous activity did not (B = 0.69, *t* = 0.981, *p* = 0.328). Gender was significant, again indicating more creative achievements for women than men (B = -20.04, *t* = -3.308, *p* = 0.001). Age (B = 0.04, *t* = 0.072, *p* = 0.942), BMI (B = 1.06, *t* = 1.417, *p* = 0.159), and openness (B = 3.52, *t* = 0.582, *p* = 0.561) were not significant. The RIBS (B = 13.65, *t* = 3.723, *p* < 0.001) and the creative potential (TCT-DP; B = 10.92, *t* = 2.956, *p* = 0.004) were significant and independent predictors of creative achievement (see Table 3).

The results were similar when using sedentariness or steps per minute in the regression analyses, although steps per minute only reach a *p* = 0.060 (see supplementary Table 1 and Table 2). Poisson regression models, which account for skewed distributions, confirmed all significant predictors of real-life creativity.

Furthermore, the separate Pearson correlations between the CAct subscales and measures of objective moderate-to-vigorous PA were significant for cooking, music, and sports (see supplementary Table 3). Similarly subjective PA was significantly associated with the subscales cooking, sports, as well as science and engineering. For creative achievements, only cooking was associated with objective PA, while sports, performing arts, as well as science and engineering were correlated with subjective PA (see supplementary Table 4). When calculating separate correlations for the subjective PA subscales (i.e., everyday, leisure time, and sports METs), we found that the significant findings are largely due to everyday and leisure time activities but not due to sports (see supplementary Tables 3 and 4).

## Discussion

This study explored new avenues by investigating if naturally occurring habitual PA is associated with real-life creative activities and achievements in a cross-sectional study. To the best of our knowledge, this study provides first evidence that higher PA of moderate-to-vigorous intensity as well as more steps per minute and lower sedentariness are associated with more frequent engagement in creative activities, especially in the domains of cooking, sports, and music. Of note, we observed a similar pattern of results for self-rated habitual PA (i.e., total METs). Together, these findings indicate that more physically active people show more real-life creative behavior, which extends the literature indicating positive effects of PA on creative ideation performance on a chronical^44^ as well as acute level^45^ in children and in adolescent (for a meta-analyses, see^15,17^).

Despite the need for replication, the relationships of objective as well as subjective PA measures with real-life creativity seem plausible because of the following reasons. Firstly, regular PA improves cognitive functions, such as memory, attention, and executive function^13^. These cognitive enhancements can foster creative ideation performance^15^, and thereby support engagement in creative activities (as well as more creative achievements; see e.g.,^24^). This aligns with embodied perspectives on cognition, which argues that bodily movements play a key role in the development of creative ideas^46^. In line with this, Oppezzo and Schwartz^47^ found that walking improves the quality of (verbal) ideas. Similarly, Bollimbala et al.^48^ indicated that enjoyable PA such as dancing showed acute and longer lasting enhancing effects on divergent thinking skills^49^. Future studies should include additional measures of creative ideation performance as well as basic cognitive functions to enable a more in-depth examination of the observed effects.

Secondly, moderate-to-vigorous activity stimulates brain regions related to creative ideation (e.g.,^50^). Beyond function, PA also impacts the structure of the brain^51^. Fink et al.^6^ found an increase in hippocampal volume, which was accompanied with changes in affective states. In line with this, PA seems to stimulate the release of neurotransmitters playing a key role for affect, cognition, and creative abilities and achievements such as dopamine^52–54^. Interestingly, research indicated a relationship of PA with openness, the personality trait referring to the tendency to be creative and curious^55,56^. In this regard, Lydon-Staley^57^ reported higher curiosity when people show more PA. Since we did not find an association between openness and PA in this study, it is possible that relationships might be specific to curiosity and dopamine. Accordingly, future investigations should additionally measure these variables to get a deeper insight into potential mechanisms^58^.

Thirdly, related to the previous arguments, PA could improve positive affect and reduce negative affect, anxiety, depression, and stress^59–63^. This affective impact of PA might free up resources to think more creatively and therefore, increase engagement in more enjoyable creative activities ultimately leading to more creative achievements^64^. Although positive affect seems not to be a strong mechanism for the association between PA and creative ideation performance^21,22,65,66^, it might play a more important role for creative activities and achievements. Accordingly, ambulatory and daily dairy studies showed that positive affect was associated with creative activities in everyday life (^67–70^; or vice versa;^71^). Because the present study focused on habitual relationships and therefore neglected to assess the dynamics of PA and real-life creativity from day to day and from moment to moment, future studies should evaluate if positive affect mediates the relation between PA and real-life creative activities as well as achievements more dynamically (see e.g.,^70,72,73^ for a dynamic assessment of creative activities). This would also allow us to shed more light on the causal mechanisms by testing cross-lagged relationships. Furthermore, future replications should include the amount of enjoyable PA^48,49^, which might lead to the observation of a more robust relationship.

Fourth, the association between PA and real-life creative activities fits the ideas of Kaufman^74^, who suggested that creativity can serve the function to find meaning in life. In line with this, Hooker et al.^75^ reported a positive link between PA and the purpose in life (for similar findings in an older sample see^76^). Hooker et al.^75^ hypothesized that people with a higher purpose in life also target to maintain their health and consequently show health promoting behavior such as PA. In this respect, perceived meaning and purpose in life might link real-life creative activities (as well as achievements) with PA. To investigate this in more detail, future research should additionally assess purpose in life and associated variables such as perceived meaning, as well as well-being, depression, and stress to identify third hidden variables impacting both PA and creativity. In this context, self-regulatory strength might be a further powerful candidate to explain why more PA and more creative activities are associated with each other^77^.

Contrary to our expectations, we did not find an association between PA and self-reported creative ideational behavior (RIBS) as well as people’s creative potential in the figural domain (TCT-DP; but see^20,78,79^). At the first view, these null findings seem to disagree with previous research; however, a closer look suggests that the pattern of findings aligns with the literature. Firstly, Top and Akil^80^ reported no effects for the association between PA and self-reported creative behavior (for negative relationship see^81^); however, evidence is very scarce. Secondly, previous research reported different effects of PA for verbal and figural creative ideation performance, showing no cross-sectional and direct association between PA and figural creative ideation^22^. Furthermore, Piya-Amornphan et al.^78^ only reported a small significant relationship between PA and creative ability in one out of three age groups (i.e., 14–17), whereas they did not find a link in children between 6 and 9 years and 10 and 13 years. Thirdly, Piya-Amornphan et al.^20^ reported an association between figural originality, fluency, and flexibility (but not elaboration) with moderate-to-vigorous PA. However, they used the figural version of the TTCT in a young sample (10–11 years) and indexed originality as a frequency-based sum-score^82,83^. Additionally, the present study assessed creative ability in the figural domain via an online application of the TCT-DP, not allowing a strict control (e.g., timing). However, since we found the expected pattern of associations for both the RIBS and the TCT-DP with real-life creative achievements and creative activities, as well as openness in this study, the applied methods seem valid. To sum up, the present results argue for the assumption that the association between habitual PA and figural creative ideation performance seems to be weak^22^. More rigorous studies considering domain specificity in creative ability may help to shed further light on these relationships.

Interestingly, the present study found no (linear) association between creative achievements and objective PA (although the association emerged with subjective PA), suggesting that while PA may enhance immediate creative thinking^15,48^, it may not that strongly influence long-term real-life creative achievements. This reduced effect size could indicate that while PA might boost creative abilities it might not that strongly impact other essential components of creativity, such as domain knowledge or sustained motivation in the same way^84^. Additionally, time or energy dedicated to PA might detract from the time required for creative pursuits, resulting in little net gain in real-world creative achievements.

### Limitations

Although objective assessment of physical activity via sensors has numerous advantages^20^, we still need to acknowledge that we assessed the relationship between PA and creative activities at a cross-sectional level. Therefore, in contrast to randomized controlled trials (see e.g.,^44^), we do not know the causal direction of effects. For example, we cannot decide if creative activities and achievements increase PA or vice versa. Both directions are plausible to some extent. Nevertheless, this study adds further evidence on the positive association between PA and creativity and has the clear strength of measuring both objective and subjective PA^85^. Most of the previous research only applied one of both methods to assess habitual PA and often was limited to creative ideation performance (see e.g.,^36,86^ for using questionnaires) and thus disregarding real-life creative behavior. The combined use of subjective and objective PA measures allowed for a more comprehensive investigation of the hypothesized relationship. Convincingly, both methods showed a coherent pattern of findings, that is, higher PA was linked to more real-life creativity, and they even explained variance in creative behavior beyond the more classical variables such as openness, and creative potential in the figural domain. Notably, both subjective and objective measures of PA showed only a weak association with each other. One potential explanation is that a five-day assessment might not capture all levels of bodily movement, which might be better reflected in a questionnaire (e.g., hiking twice a month). However, at the same time questionnaires additionally assess self-beliefs and heuristics about once own PA levels, thus adding further independent and meaningful variance to this self-rating measure. Therefore, although at the lower boundary, the size of the obtained relationship is in line with literature^85,87,88^.

Remarkably, in the present study, creative activities and achievements of cooking and sports are inherently linked with PA. With the current data, it is difficult to disentangle the source of these associations in terms of determining if they simply emerged because the creative activities of sports and cooking require PA per se. However, we want to highlight that we also observed associations of PA with making music, science and engineering, and performing arts. Furthermore, when looking deeper into the data, self-rated sports activities were not linked with creative achievements at all (but everyday activities and leisure time activities were). Nevertheless, it is an intriguing objective to disentangle the reasons for and consequences of PA with a more dynamic approach to real-life behavior as outlined above.

## Conclusions

This study is (among) the first that investigated the association between PA and real-life creative behaviors. In line with our assumptions, we found positive relationships between PA and creative activities as well as achievements. This extends the knowledge in the rich research field on creativity and PA and shows that PA is not only associated with health variables, basic cognitions, and more complex cognitive functions such as the creative ideation process but also relates to the daily routines of doing something creative. Consequently, PA might not only change high level cognitive functioning—that is the production of a creative idea when asked to find an original use of an everyday object—but may also have the potential to impact our daily life creative behaviors and the content of our daily routines and activities.

## Electronic supplementary material

Below is the link to the electronic supplementary material.

Supplementary Material 1

## Data Availability

The datasets generated during and/or analysed during the current study are available from the corresponding author.
