# Supplementary material for: Habitual physical activity is related to more creative activities and achievements
Source: Sci Rep. 2024 Nov 30;14:29768. doi: 10.1038/s41598-024-80714-6 (PMC11607317; doi:10.1038/s41598-024-80714-6)
Supplement: Supplementary file 1 — Supplementary Material 1 [file 41598_2024_80714_MOESM1_ESM.docx]

Supplementary Table 1. Regression analysis predicting CAct and CAch via Sedentariness

|  | **CAct** | | | **CAch** | | |
| --- | --- | --- | --- | --- | --- | --- |
| *Predictors* | *Estimates* | *Conf. Int (95%)* | *p-value* | *Estimates* | *Conf. Int (95%)* | *p-value* |
| Intercept | 59.87 | 8.80 – 110.94 | **0.022** | -28.29 | -109.46 – 52.88 | 0.492 |
| Gender | -15.62 | -22.99 – -8.25 | **<0.001** | -19.48 | -31.19 – -7.76 | **0.001** |
| Age | -0.44 | -1.16 – 0.28 | 0.229 | -0.03 | -1.17 – 1.12 | 0.960 |
| BMI | -0.57 | -1.50 – 0.35 | 0.223 | 1.04 | -0.44 – 2.51 | 0.166 |
| Openness | 5.83 | -1.67 – 13.33 | 0.127 | 3.87 | -8.05 – 15.79 | 0.522 |
| RIBS | 10.04 | 5.48 – 14.60 | **<0.001** | 13.65 | 6.41 – 20.89 | **<0.001** |
| TCT-DP | 3.05 | -1.54 – 7.64 | 0.191 | 10.91 | 3.62 – 18.20 | **0.004** |
| Objective PA (Sedentariness) | -0.52 | -0.98 – -0.07 | **0.025** | -0.39 | -1.11 – 0.33 | 0.289 |
| Subjective PA (total METs) | 0.12 | 0.03 – 0.22 | **0.013** | 0.17 | 0.02 – 0.33 | **0.030** |
| Observations | 156 | | | 156 | | |
| R^2^ / R^2^ adjusted | 0.345 / 0.310 | | | 0.272 / 0.232 | | |

*Note.* Gender was coded 0 = women and 1 = men. BMI = body mass index.

Supplementary Table 2. Regression analysis predicting CAct and CAch via Steps

|  | **CAct** | | | **CAch** | | |
| --- | --- | --- | --- | --- | --- | --- |
| *Predictors* | *Estimates* | *Conf. Int (95%)* | *p-value* | *Estimates* | *Conf. Int (95%)* | *p-value* |
| Intercept | 13.03 | -21.71 – 47.78 | 0.460 | -61.55 | -116.69 – -6.41 | **0.029** |
| Gender | -14.68 | -22.01 – -7.34 | **<0.001** | -18.63 | -30.27 – -6.99 | **0.002** |
| Age | -0.40 | -1.13 – 0.32 | 0.270 | 0.03 | -1.11 – 1.18 | 0.958 |
| BMI | -0.59 | -1.52 – 0.34 | 0.212 | 1.01 | -0.47 – 2.49 | 0.178 |
| Openness | 5.40 | -2.17 – 12.96 | 0.161 | 3.84 | -8.16 – 15.84 | 0.528 |
| RIBS | 10.04 | 5.46 – 14.62 | **<0.001** | 13.71 | 6.44 – 20.98 | **<0.001** |
| TCT-DP | 2.83 | -1.77 – 7.44 | 0.226 | 10.72 | 3.41 – 18.03 | **0.004** |
| Steps | 0.82 | -0.03 – 1.68 | 0.060 | 0.21 | -1.15 – 1.57 | 0.760 |
| Total METs | 0.13 | 0.04 – 0.23 | **0.007** | 0.18 | 0.03 – 0.34 | **0.019** |
| Observations | 156 | | | 156 | | |
| R^2^ / R^2^ adjusted | 0.339 / 0.303 | | | 0.267 / 0.227 | | |

*Note.* Gender was coded 0 = women and 1 = men. BMI = body mass index.

Supplementary Table 3. Pearson correlations among variables for the CAct subscales

|  | *Everyday METs* | *Leisure METs* | *Sport METs* | *Steps* | *Sedentariness* | *Moderate-to-vigorous PA* | *Literature* | *Music* | *Arts and crafts* | *Cooking* | *Sports* | *Visual arts* | *Performing arts* | *Science and engineering* |
| --- | --- | --- | --- | --- | --- | --- | --- | --- | --- | --- | --- | --- | --- | --- |
| *Subjective PA (total METs)* | 0.416 *(<.001)* | 0.508 *(<.001)* | 0.796 *(<.001)* | 0.148 *(.065)* | -0.211 *(.008)* | 0.164 *(.040)* | 0.120 *(.134)* | 0.069 *(.392)* | 0.091 *(.261)* | 0.288 *(<.001)* | 0.214 *(.007)* | 0.077 *(.340)* | 0.079 *(.326)* | 0.185 *(.021)* |
| *Everyday METs* |  | 0.412 *(<.001)* | -0.102 *(.205)* | 0.061 *(.451)* | -0.073 *(.367)* | 0.088 *(.274)* | 0.092 *(.253)* | 0.030 *(.710)* | 0.225 *(.005)* | 0.223 *(.005)* | 0.078 *(.332)* | 0.213 *(.008)* | 0.194 *(.016)* | 0.173 *(.030)* |
| *Leisure METs* | 0.412 *(<.001)* |  | -0.012 *(.878)* | 0.109 *(.176)* | -0.110 *(.173)* | 0.080 *(.321)* | 0.085 *(.292)* | 0.062 *(.442)* | 0.161 *(.045)* | 0.220 *(.006)* | 0.144 *(.073)* | 0.224 *(.005)* | 0.157 *(.051)* | 0.138 *(.086)* |
| *Sport METs* | -0.102 *(.205)* | -0.012 *(.878)* |  | 0.103 *(.199)* | -0.173 *(.031)* | 0.124 *(.123)* | 0.068 *(.400)* | 0.042 *(.601)* | -0.057 *(.478)* | 0.154 *(.055)* | 0.158 *(.048)* | -0.097 *(.228)* | -0.056 *(.488)* | 0.088 *(.275)* |
| *Steps* | 0.061 *(.451)* | 0.109 *(.176)* | 0.103 *(.199)* |  | -0.780 *(<.001)* | 0.905 *(<.001)* | -0.006 *(.946)* | 0.135 *(.092)* | 0.070 *(.386)* | 0.191 *(.017)* | 0.259 *(.001)* | 0.033 *(.682)* | 0.123 *(.125)* | 0.118 *(.142)* |
| *Sedentariness* | -0.073 *(.367)* | -0.110 *(.173)* | -0.173 *(.031)* | -0.780 *(<.001)* |  | -0.826 *(<.001)* | 0.033 *(.679)* | -0.178 *(.026)* | -0.054 *(.507)* | -0.149 *(.063)* | -0.307 *(<.001)* | -0.005 *(.946)* | -0.007 *(.927)* | -0.116 *(.149)* |
| *Moderate-to-vigorous PA* | 0.088 *(.274)* | 0.080 *(.321)* | 0.124 *(.123)* | 0.905 *(<.001)* | -0.826 *(<.001)* |  | -0.022 *(.783)* | 0.162 *(.044)* | 0.017 *(.835)* | 0.178 *(.026)* | 0.279 *(<.001)* | -0.012 *(.886)* | 0.059 *(.461)* | 0.128 *(.111)* |
| *Literature* | 0.092 *(.253)* | 0.085 *(.292)* | 0.068 *(.400)* | -0.006 *(.946)* | 0.033 *(.679)* | -0.022 *(.783)* |  | 0.326 *(<.001)* | 0.277 *(<.001)* | 0.226 *(.005)* | 0.070 *(.383)* | 0.380 *(<.001)* | 0.355 *(<.001)* | 0.138 *(.085)* |
| *Music* | 0.030 *(.710)* | 0.062 *(.442)* | 0.042 *(.601)* | 0.135 *(.092)* | -0.178 *(.026)* | 0.162 *(.044)* | 0.326 *(<.001)* |  | 0.183 *(.022)* | 0.337 *(<.001)* | 0.139 *(.085)* | 0.297 *(<.001)* | 0.303 *(<.001)* | 0.145 *(.071)* |
| *Arts and crafts* | 0.225 *(.005)* | 0.161 *(.045)* | -0.057 *(.478)* | 0.070 *(.386)* | -0.054 *(.507)* | 0.017 *(.835)* | 0.277 *(<.001)* | 0.183 *(.022)* |  | 0.616 *(<.001)* | 0.170 *(.034)* | 0.633 *(<.001)* | 0.456 *(<.001)* | 0.099 *(.217)* |
| *Cooking* | 0.223 *(.005)* | 0.220 *(.006)* | 0.154 *(.055)* | 0.191 *(.017)* | -0.149 *(.063)* | 0.178 *(.026)* | 0.226 *(.005)* | 0.337 *(<.001)* | 0.616 *(<.001)* |  | 0.213 *(.008)* | 0.491 *(<.001)* | 0.339 *(<.001)* | 0.234 *(.003)* |
| *Sports* | 0.078 *(.332)* | 0.144 *(.073)* | 0.158 *(.048)* | 0.259 *(.001)* | -0.307 *(<.001)* | 0.279 *(<.001)* | 0.070 *(.383)* | 0.139 *(.085)* | 0.170 *(.034)* | 0.213 *(.008)* |  | 0.197 *(.013)* | 0.296 *(<.001)* | 0.208 *(.009)* |
| *Visual arts* | 0.213 *(.008)* | 0.224 *(.005)* | -0.097 *(.228)* | 0.033 *(.682)* | -0.005 *(.946)* | -0.012 *(.886)* | 0.380 *(<.001)* | 0.297 *(<.001)* | 0.633 *(<.001)* | 0.491 *(<.001)* | 0.197 *(.013)* |  | 0.447 *(<.001)* | 0.153 *(.056)* |
| *Performing arts* | 0.194 *(.016)* | 0.157 *(.051)* | -0.056 *(.488)* | 0.123 *(.125)* | -0.007 *(.927)* | 0.059 *(.461)* | 0.355 *(<.001)* | 0.303 *(<.001)* | 0.456 *(<.001)* | 0.339 *(<.001)* | 0.296 *(<.001)* | 0.447 *(<.001)* |  | 0.172 *(.032)* |
| *Science and engineering* | 0.173 *(.030)* | 0.138 *(.086)* | 0.088 *(.275)* | 0.118 *(.142)* | -0.116 *(.149)* | 0.128 *(.111)* | 0.138 *(.085)* | 0.145 *(.071)* | 0.099 *(.217)* | 0.234 *(.003)* | 0.208 *(.009)* | 0.153 *(.056)* | 0.172 *(.032)* |  |

Note. *p* values in parentheses. PA METs = Sum of METs per week assessed via FQPA.

Supplementary Table 4. Pearson correlations among variables for the CAch subscales

|  | *Everyday METs* | *Leisure METs* | *Sport METs* | *Steps* | *Sedentariness* | *Moderate-to-vigorous PA* | *Literature* | *Music* | *Arts and crafts* | *Cooking* | *Sports* | *Visual arts* | *Performing arts* | *Science and engineering* |
| --- | --- | --- | --- | --- | --- | --- | --- | --- | --- | --- | --- | --- | --- | --- |
| *Subjective PA (total METs)* | 0.416 *(<.001)* | 0.508 *(<.001)* | 0.796 *(<.001)* | 0.148 *(.065)* | -0.211 *(.008)* | 0.164 *(.040)* | 0.011 *(.891)* | 0.032 *(.695)* | 0.065 *(.417)* | 0.125 *(.119)* | 0.203 *(.011)* | 0.086 *(.288)* | 0.199 *(.013)* | 0.176 *(.028)* |
| *Everyday METs* |  | 0.412 *(<.001)* | -0.102 *(.205)* | 0.061 *(.451)* | -0.073 *(.367)* | 0.088 *(.274)* | 0.161 *(.044)* | 0.096 *(.231)* | 0.214 *(.007)* | 0.172 *(.031)* | 0.150 *(.061)* | 0.258 *(.001)* | 0.345 *(<.001)* | 0.246 *(.002)* |
| *Leisure METs* | 0.412 *(<.001)* |  | -0.012 *(.878)* | 0.109 *(.176)* | -0.110 *(.173)* | 0.080 *(.321)* | 0.040 *(.619)* | 0.108 *(.178)* | 0.191 *(.017)* | 0.196 *(.014)* | 0.190 *(.018)* | 0.263 *(.001)* | 0.289 *(<.001)* | 0.240 *(.003)* |
| *Sport METs* | -0.102 *(.205)* | -0.012 *(.878)* |  | 0.103 *(.199)* | -0.173 *(.031)* | 0.124 *(.123)* | -0.072 *(.373)* | -0.051 *(.529)* | -0.096 *(.233)* | -0.010 *(.906)* | 0.095 *(.236)* | -0.123 *(.126)* | -0.034 *(.672)* | 0.000 *(.997)* |
| *Steps* | 0.061 *(.451)* | 0.109 *(.176)* | 0.103 *(.199)* |  | -0.780 *(<.001)* | 0.905 *(<.001)* | -0.092 *(.253)* | 0.057 *(.481)* | 0.059 *(.464)* | 0.169 *(.034)* | 0.035 *(.663)* | -0.027 *(.735)* | 0.120 *(.137)* | 0.104 *(.198)* |
| *Sedentariness* | -0.073 *(.367)* | -0.110 *(.173)* | -0.173 *(.031)* | -0.780 *(<.001)* |  | -0.826 *(<.001)* | 0.073 *(.364)* | -0.103 *(.202)* | 0.008 *(.921)* | -0.164 *(.041)* | -0.124 *(.122)* | 0.030 *(.712)* | -0.066 *(.414)* | -0.126 *(.117)* |
| *Moderate-to-vigorous PA* | 0.088 *(.274)* | 0.080 *(.321)* | 0.124 *(.123)* | 0.905 *(<.001)* | -0.826 *(<.001)* |  | -0.096 *(.235)* | 0.111 *(.167)* | 0.039 *(.627)* | 0.195 *(.015)* | 0.031 *(.701)* | -0.053 *(.507)* | 0.095 *(.240)* | 0.125 *(.120)* |
| *Literature* | 0.161 *(.044)* | 0.040 *(.619)* | -0.072 *(.373)* | -0.092 *(.253)* | 0.073 *(.364)* | -0.096 *(.235)* |  | 0.171 *(.033)* | 0.031 *(.701)* | 0.055 *(.497)* | 0.146 *(.069)* | 0.305 *(<.001)* | 0.300 *(<.001)* | 0.229 *(.004)* |
| *Music* | 0.096 *(.231)* | 0.108 *(.178)* | -0.051 *(.529)* | 0.057 *(.481)* | -0.103 *(.202)* | 0.111 *(.167)* | 0.171 *(.033)* |  | 0.069 *(.391)* | 0.173 *(.031)* | 0.076 *(.346)* | 0.086 *(.288)* | 0.313 *(<.001)* | 0.020 *(.808)* |
| *Arts and crafts* | 0.214 *(.007)* | 0.191 *(.017)* | -0.096 *(.233)* | 0.059 *(.464)* | 0.008 *(.921)* | 0.039 *(.627)* | 0.031 *(.701)* | 0.069 *(.391)* |  | 0.372 *(<.001)* | 0.283 *(<.001)* | 0.428 *(<.001)* | 0.187 *(.019)* | 0.297 *(<.001)* |
| *Cooking* | 0.172 *(.031)* | 0.196 *(.014)* | -0.010 *(.906)* | 0.169 *(.034)* | -0.164 *(.041)* | 0.195 *(.015)* | 0.055 *(.497)* | 0.173 *(.031)* | 0.372 *(<.001)* |  | 0.173 *(.031)* | 0.247 *(.002)* | 0.255 *(.001)* | 0.265 *(.001)* |
| *Sports* | 0.150 *(.061)* | 0.190 *(.018)* | 0.095 *(.236)* | 0.035 *(.663)* | -0.124 *(.122)* | 0.031 *(.701)* | 0.146 *(.069)* | 0.076 *(.346)* | 0.283 *(<.001)* | 0.173 *(.031)* |  | 0.305 *(<.001)* | 0.301 *(<.001)* | 0.454 *(<.001)* |
| *Visual arts* | 0.258 *(.001)* | 0.263 *(.001)* | -0.123 *(.126)* | -0.027 *(.735)* | 0.030 *(.712)* | -0.053 *(.507)* | 0.305 *(<.001)* | 0.086 *(.288)* | 0.428 *(<.001)* | 0.247 *(.002)* | 0.305 *(<.001)* |  | 0.380 *(<.001)* | 0.360 *(<.001)* |
| *Performing arts* | 0.345 *(<.001)* | 0.289 *(<.001)* | -0.034 *(.672)* | 0.120 *(.137)* | -0.066 *(.414)* | 0.095 *(.240)* | 0.300 *(<.001)* | 0.313 *(<.001)* | 0.187 *(.019)* | 0.255 *(.001)* | 0.301 *(<.001)* | 0.380 *(<.001)* |  | 0.324 *(<.001)* |
| *Science and engineering* | 0.246 *(.002)* | 0.240 *(.003)* | 0.000 *(.997)* | 0.104 *(.198)* | -0.126 *(.117)* | 0.125 *(.120)* | 0.229 *(.004)* | 0.020 *(.808)* | 0.297 *(<.001)* | 0.265 *(.001)* | 0.454 *(<.001)* | 0.360 *(<.001)* | 0.324 *(<.001)* |  |

Note. *p* values in parentheses. PA METs = Sum of METs per week assessed via FQPA.
